# *Chlamydomonas* as a model for reactive oxygen species signaling and thiol redox regulation in the green lineage

**DOI:** 10.1093/plphys/kiab355

**Published:** 2021-08-30

**Authors:** Setsuko Wakao, Krishna K. Niyogi

**Affiliations:** 1 Division of Molecular Biophysics and Integrated Bioimaging, Lawrence Berkeley National Laboratory, Berkeley, California 94720, USA; 2 Department of Plant and Microbial Biology, University of California, Berkeley, California 94720, USA; 3 Howard Hughes Medical Institute, University of California, Berkeley, California 94720, USA

## Abstract

One-sentence summary: Advances in proteomic and transcriptomic studies have made *Chlamydomonas* a powerful research model in redox and reactive oxygen species regulation with unique and overlapping mechanisms with plants.

While the maintenance of redox balance is important for all organisms, the energy balance in the chloroplast is particularly tightly linked to the overall cellular redox status in photosynthetic organisms. Excess light can cause overreduction of the photosynthetic electron transport chain, which, when unchecked, results in the generation of reactive oxygen species (ROS) that can oxidize cellular components including nucleic acids, proteins, and lipids (Li et al., 2009). A photosynthetic cell possesses multiple layers of defense against oxidative stress, such as antioxidant enzymes (Foyer and Noctor, 2005) and alternative electron transfer pathways (Alric and Johnson, 2017). At the same time, ROS can function as important signals that mediate responses to biotic and abiotic stresses (Apel and Hirt, 2004). Reversibly oxidizable small molecules and proteins can serve as ROS sinks and signals and are re-reduced by multiple mechanisms. For example, the ascorbate–glutathione (GSH) cycle is central in controlling ROS levels and regenerating/reducing oxidized cellular redox agents (Figure 1A; reviewed by Foyer and Noctor, 2011). The functions of many proteins can be regulated by the redox state of cysteine residues, such as the formation or reduction of disulfides by thioredoxins (TRX) (Buchanan, 1991) and glutaredoxins (Michelet et al., 2013b; Figure 1, B and C).

**Figure 1:**
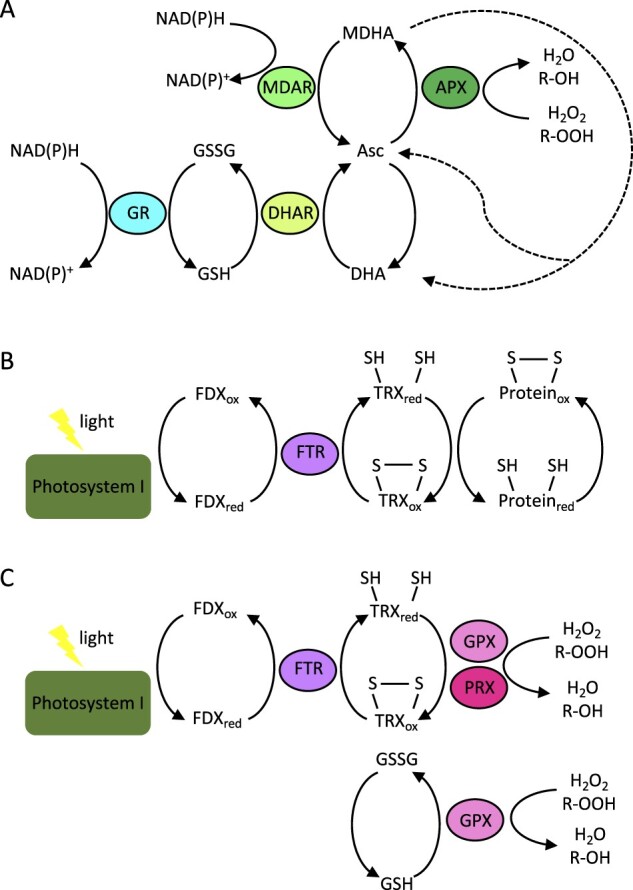
Redox reactions involving ascorbate–glutathione cycle and TRX. A, The reduction–oxidation cycle of ascorbate serves as cofactor for many enzymes such as APX, a major peroxide detoxifying enzyme under light stress. The regeneration of Asc from its oxidized form utilizes the enzymes MDAR and DHAR, which are coupled to the oxidation of NADPH and glutathione, respectively. The regeneration of GSH is catalyzed by GR coupled to NADPH. Dashed arrows represent the interconversion of Asc, MDHA, DHA that occurs without enzymatic action. B, C, Light-driven reduction of ferredoxin (FDX) is transduced through FTR to TRX. (B) The TRX-redox cycle is used for reduction and oxidation of disulfides and (C) coupled to the detoxification of peroxides by enzymes such as GPX and PRX. Text in black is compounds in their reduced or oxidized forms (indicated by suffixes: red, reduced; ox, oxidized). Asc, ascorbate; MDHA, monodyhydroascorbate; DHA, dehydroascorbate; GSH, reduced glutathione; GSSG, glutathione disulfide; NADPH, nicotinamide adenine dinucleotide phosphate. Colored circles are enzymes with oxido-reductase activity. GR, glutathione reductase; FRX, Ferredoxin; FTR, ferredoxin–thioredoxin reductase.

For more than half a century, the green alga *Chlamydomonas* (*Chlamydomonas reinhardtii*) has been a model organism in which mutants can be generated to study biological processes such as photosynthesis (Levine, 1960). As described in this update review, *Chlamydomonas* has metabolic and molecular pathways that are analogous to those in the model plant Arabidopsis (*Arabidopsis thaliana*) and those that are distinct, offering a glimpse into the diversity of photosynthetic organisms. *Chlamydomonas* has several advantages as an experimental organism for studying redox regulation and ROS signaling. Because it is unicellular, *Chlamydomonas* can be grown in uniform cultures, and ROS induction and treatment are relatively easy compared to working with whole, multicellular plants. These strengths have been leveraged to generate a wealth of RNA-Seq and proteomics datasets that brings us closer to a systems-level understanding of redox regulation. This update review discusses the recent advances toward understanding cellular responses to ROS and redox perturbation in *Chlamydomonas*. Numerous recent studies have highlighted the importance of the ascorbate–GSH cycle in redox homeostasis in *Chlamydomonas* as well as ascorbate’s indirect roles in photoprotection. The relatively fewer variables in experimental conditions have facilitated cross-comparison of transcriptomic responses to different redox-active or photosensitizing molecules that generate ROS and uncovered unique physiological responses in *Chlamydomonas* as compared to plants. Proteomic experiments have revealed the striking fact that the Calvin–Benson (CB) cycle is targeted by multiple post-translational modifications (PTMs), including enzymes already known to be subject to redox regulation, and what future work is needed in this area will be discussed.

## ROS and photosynthesis

**Figure 2:**
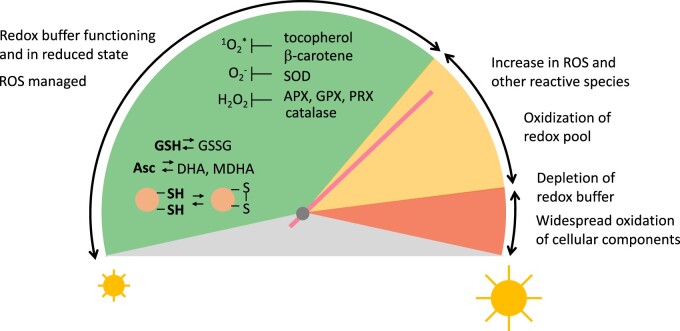
A speedometer illustration to represent the transition of a healthy cell to a cell that is experiencing oxidative stress. In a healthy cell under normal light intensity, the ascorbate–glutathione cycle and redox proteins constantly carry out redox reactions to maintain redox homeostasis. Generation of ROS is also part of a healthy cell metabolism, which is kept under control by various mechanisms shown. As oxidation takes over in a stressed cell, for example under excess light, the redox balance shifts toward oxidized (yellow). Increases in ROS and oxidation of the redox pool occur, which further develop into the depletion of redox buffer and widespread oxidation of cellular components (red). Under oxidative stress, the cell can respond, putting the brakes on oxidation, and the cell can still return to a healthy state even after reaching the red zone. Asc, ascorbate; MDHA, monodyhydroascorbate; DHA, dehydroascorbate; GSH, reduced glutathione; GSSG, glutathione disulfide; ^1^O_2_*, singlet oxygen; $O_{2}^{-}$, superoxide.

Photosynthetic organisms must acclimate to dynamic changes in parameters such as light, temperature, and nutrients in the natural environment in order to optimize photosynthesis. ROS are unavoidable byproducts of oxygenic photosynthesis and could overwhelm the cellular redox equilibrium, often referred to as an oxidative burst, under conditions such as biotic or abiotic stress (Figure 2). In the last two decades, ROS have been demonstrated as important signaling molecules, rather than solely deleterious oxidative compounds. ROS are diverse, with hydrogen peroxide (H_2_O_2_), superoxide (O_2_^−^), and singlet oxygen (^1^O_2_*) being the most often discussed ROS in the context of photosynthesis. Different forms of ROS have different modes of generation, reactivities, and cellular mechanisms that deal with them (Apel and Hirt, 2004).

H_2_O_2_ is ubiquitous. It is produced in multiple subcellular compartments and through different processes, for example, in the peroxisome during photorespiration (Foyer et al., 2009) and in the chloroplast by pseudocyclic electron transport, also called the water–water cycle (Asada, 1999). H_2_O_2_ and alkyl peroxides are detoxified into H_2_O and alkyl alcohols, respectively, by enzymes such as ascorbate peroxidase (APX), glutathione peroxidase (GPX), catalase, and peroxiredoxin (PRX; reviewed by Smirnoff and Arnaud, 2019; Figure 1C). The role of H_2_O_2_ in signaling has been well established in Arabidopsis (reviewed by Dietz et al., 2016). Studies on *Chlamydomonas* mutants of H_2_O_2_-detoxifying enzymes have highlighted their importance in both protection and signaling. Catalase is one such enzyme that is transiently and reversibly inactivated upon transfer to excess light (700 µmol photons m^−2^ s^−1^) from darkness, allowing a sharp rise in H_2_O_2_ levels and a subsequent nuclear gene expression response (Shao et al., 2008; Michelet et al., 2013a). The silencing of catalase abolishes the spike in H_2_O_2_ and renders the strain photosensitive (Michelet et al., 2013a), indicating that the elevated H_2_O_2_ is not toxic but rather a necessary step to trigger an H_2_O_2_-dependent response for coping with further stress. The reversible inactivation of catalase occurs through a one-electron reduction and is hypothesized to occur via a single cysteine residue that may somehow sense overreduction of photosynthetic electron transport (Michelet et al., 2013a). GPX is another important H_2_O_2_- and alkyl peroxide-detoxifying enzyme (Fischer et al., 2009). Some GPXs occur as seleno enzymes (Se-GPX) that utilize a seleno-cysteine at their active sites, whereas others use an unmodified cysteine (nonseleno GPXs, NS-GPX). The *Chlamydomonas* genomes encode five GPXs, two Se-GPXs (GPX1 and GPX2), and three NS-GPXs (GPX3, GPX4, and GPX5), which is in contrast to land plants, which are considered to have lost the Se-GPXs as have yeast and some animals (Dayer et al., 2008). The two types of GPXs are presumed to accept electrons from different donors: TRX for NS-GPX and GSH for Se-GPX, exemplifying the various ways in which the redox system components can interact with each other (Figure 1C). The *Chlamydomonas* mutant *gpx5* exhibits photooxidative stress and is unable to accumulate lipid under nitrogen depletion (Ma et al., 2020). *GPX5* has been used extensively as a marker for studying ROS-specific gene expression in *Chlamydomonas* and will be discussed further in the ROS-specific gene expression section below.

In the chloroplast, O_2_ at the acceptor side of photosystem (PS) I is photoreduced via flavodiiron (FLV) proteins that catalyze NADPH-dependent reduction of O_2_ to H_2_O (Chaux et al., 2017) or via the Mehler reaction (Mehler, 1951), which generates O_2_^−^. The O_2_^−^ produced by the Mehler reaction is converted to H_2_O_2_ by superoxide dismutase (SOD) and subsequently to H_2_O by APX (Asada, 1999). FLVs are conserved in cyanobacteria, algae, and mosses but are absent in angiosperms (Yamamoto et al., 2016). FLV-dependent photoreduction of O_2_ in *Chlamydomonas* is prominent during dark-to-light transitions and is critical for growth in fluctuating light (Chaux et al., 2017; Jokel et al., 2018; Saroussi et al., 2019). Detoxification of O_2_^−^ is an important protective mechanism in plants as shown by the photosensitive phenotype of a knockdown of an isoform of SOD in Arabidopsis (Rizhsky et al., 2003), but its role in *Chlamydomonas* has not yet been investigated. Among the three types of SODs grouped by their metal cofactors, FeSOD has been associated with the chloroplast, MnSOD with mitochondria and peroxisomes, and Cu/ZnSOD with the chloroplast and cytosol in plants (Alscher et al., 2002). The *Chlamydomonas* genome contains six *SOD* genes; five encoding MnSODs and one encoding FeSOD. The chloroplast of *Chlamydomonas* uniquely contains a MnSOD in addition to FeSOD, and in particular MnSOD3 is induced by low Fe, Mn, and H_2_O_2_ and plays an important role in the metal sparing during metal-limiting conditions (Page et al., 2012).

A chlorophyll (Chl) molecule is excited by the absorption of a photon into singlet chlorophyll (^1^Chl*), whose major fate in photosynthesis is to transfer its excited energy to a neighboring pigment. However, when PSII reaction centers are closed such as during excess light or stress conditions, the lifetime of ^1^Chl* increases, leading to increased yield of a longer-lived triplet form of Chl (^3^Chl*) (reviewed by Krieger-Liszkay, 2005). ^3^Chl* readily transfers energy to O_2_ resulting in the generation of singlet oxygen ^1^O_2_*, and therefore ^1^O_2_* is distinct from ROS such as H_2_O_2_ and O_2_^−^ as it is not a radical (reviewed by Krieger-Liszkay, 2005; Ziegelhoffer and Donohue, 2009). ^1^O_2_* is estimated to have a reactive radius of ∼100 nm in a cell (Hatz et al., 2007) and thus is not expected to travel far from its site of generation, the reaction center of PSII, before reacting with a target. However, in contrast to this notion, its leakage into the cytosol has been observed under extreme high light intensity (3,000 µmol-photon s^−1^ m^−2^) in *Chlamydomonas* (Fischer et al., 2007).

There are no known cellular detoxifying enzymes that act directly on ^1^O_2_*, and various oxidation targets of ^1^O_2_* have been reported. Two lipophilic antioxidant molecules, tocopherol and β-carotene, are positioned at the PSII reaction center and serve as quenchers and scavengers of ^1^O_2_* (Krieger-Liszkay, 2005; Krieger-Liszkay et al., 2008; Figure 2). Tocopherol quenches ^1^O_2_* through energy transfer, but it can be irreversibly oxidized to tocopherylquinone (Krieger-Liszkay and Trebst, 2006). Oxidation of β-carotene by ^1^O_2_* produces multiple molecules, including β-cyclocitral (Ramel et al., 2012a). Overaccumulation of tocopherol rescues photosensitivity of the *Chlamydomonas* mutant *npq1 lor1*, which lacks the photoprotective carotenoids zeaxanthin and lutein (Li et al., 2012). There is overwhelming evidence that associates ^1^O_2_* with the inactivation of D1 protein and PSII (reviewed by Vass and Aro, 2007). Furthermore in cyanobacteria, ^1^O_2_* is associated not only with the oxidation of D1 protein but also the inhibition of its translation elongation (Nishiyama et al., 2004), which compounds the negative effect by slowing down repair of PSII.

As ^1^O_2_* leaves the reaction center, lipid molecules composed of unsaturated fatty acids are susceptible to oxidation by ^1^O_2_*. Among the ROS, only ^1^O_2_* and hydroxyl radicals (HO⋅) have the free energy potential to perform direct lipid peroxidation (reviewed by Farmer and Mueller, 2013). ^1^O_2_* carries out only one peroxidation reaction per molecule as compared to HO⋅ that can initiate a radical-catalyzed reaction, products of which could give rise to fragmented products that can themselves form more radicals (lipid peroxyl radicals) (reviewed by Farmer and Mueller, 2013). Despite this, the main cause of lipid peroxidation in leaves under light has been shown to occur through direct peroxidation by ^1^O_2_*, even under conditions that favor HO⋅ generation (treatment with a O_2_⋅^−^ generator or in a H_2_O_2_-overproducing catalase mutant; Triantaphylidès et al., 2008). These results indicate that an uncontrolled spread of lipid peroxides through radical chain reactions is not the main outcome of ^1^O_2_* production during photooxidative stress. However, there is a significant overlap between reactive electrophile species (RES)-induced signaling and that by ^1^O_2_* in *Chlamydomonas* as will be discussed below. In the thylakoid membrane is another effective ^1^O_2_* scavenger, plastoquinol (Kruk and Trebst, 2008; Nowicka and Kruk, 2012) in addition to tocopherol (Li et al., 2012). A recent study using Arabidopsis mutants disrupted in the biosynthetic pathway of three lipophilic antioxidants, tocopherol, plastoquinone, and plastochromanol, indicates plastoquinone and tocopherol as ^1^O_2_* scavengers and plastochromanol as that for lipid peroxides under excess light (Gruszka et al., 2008; Szymańska et al., 2014; Kumar et al., 2020). Migrating further away from PSII, the γ-subunit of the chloroplast ATP synthase is known to be heavily oxidized under ^1^O_2_*-generating conditions resulting in a decrease in its proton pumping function (Mahler et al., 2007; Buchert and Forreiter, 2010). A point mutation in the CF_1_CF_o_ domain in *Chlamydomonas* ATP synthase renders it more resistant to photooxidative stress and inactivation by H_2_O_2_ in vitro but the same has not been demonstrated for ^1^O_2_* (Buchert et al., 2017).

EX1 and EX2 are required for ^1^O_2_* signaling in Arabidopsis, and EX1 has been shown to reside in the grana margins (Wagner et al., 2004; Lee et al., 2007). An oxidation at one of the two conserved Trp resides in the DUF3506 domain is required for the determination of EX1 stability and the signaling role in response to ^1^O_2_* (Dogra et al., 2019). The *Chlamydomonas* genome contains a single gene (Cre03.g163500) encoding a protein with a DUF3506 domain, which does not contain the conserved Trp residues targeted for oxidation. A suppressor screen of Arabidopsis *flu ex1* double mutant identified SAFE1 as a negative-regulator of ^1^O_2_*-induced cell death that is independent of the EX1 pathway (Wang et al., 2020). SAFE1 has a methyltransferase domain and is most similar to RMT2 (annotated as a Rubisco methyltransferase) in *Chlamydomonas*; *rmt2* mutants have been identified with varying degrees of light-sensitive and acetate-requiring phenotypes (Wakao et al., 2021). β-cyclocitral and EX1 are the only two oxidized targets demonstrated to be required for signaling in response to ^1^O_2_* in Arabidopsis. A study on RES in response to ^1^O_2_* showed that β-cyclocitral levels do not change under ROS-promoting conditions (Roach et al., 2017), suggesting that *Chlamydomonas* may not utilize the same ^1^O_2_* response pathways as Arabidopsis.

## Species-specific ROS signaling

Because multiple ROS are generated simultaneously under oxidative stress, it has been a longstanding challenge for researchers to tease apart responses that are specific to the type of ROS. There are several examples of ROS-specific regulators, namely for H_2_O_2_ and O_2_^−^, in nonphotosynthetic organisms. The bacterial protein OxyR is oxidized by H_2_O_2_, forming a disulfide bridge to activate and repress target genes (Storz et al., 1990; Zheng et al., 1998). The *Escherichia* *coli* transcriptional regulator SoxR contains an Fe-S cluster that is oxidized by O_2_^−^ to activate transcription of SoxS, which then upregulates the rest of the O_2_^−^ regulon (reviewed by Imlay, 2015). In yeast, the formation of a disulfide in a bZIP transcription factor, Yap1, is required for its transcriptional regulation activity, and this oxidation is mediated by Gpx3 that is directly oxidized by H_2_O_2_ (Delaunay et al., 2000, 2002). In yeast, the physiological response to O_2_^−^ is distinct from that for H_2_O_2_, though the O_2_^−^-specific transcriptional regulator is still unknown (Flattery-O’Brien et al., 1993; Jamieson et al., 1996).

**Figure 3:**
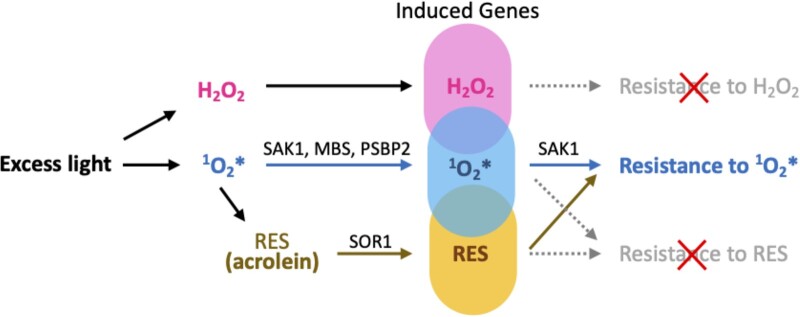
ROS- and RES-induced gene expression and acclimation in *Chlamydomonas*. Treatments with H_2_O_2_ (pink), ^1^O_2_* (blue), and acrolein (yellow) have been shown to induce the expression of distinct sets of genes with some overlaps, as shown by the colored circles. Pretreatment with ^1^O_2_* induces resistance to subsequent challenge with ^1^O_2_* (acclimation), which requires SAK1 (blue arrows). Pretreatment with acrolein induces cross-acclimation to ^1^O_2_* (brown arrows). Acclimation to H_2_O_2_ or RES has not been observed (gray dashed arrows and red X’s). Pretreatment with ^1^O_2_* does not induce cross-acclimation to RES (*t-*BOOH, Ledford et al., 2007). SAK1, METHYLENE BLUE SENSITIVE, and PSBP2 are required for ^1^O_2_*-responsive gene expression but whether these three components are part of the same or different pathways is unknown. SOR1 is a bZIP transcription factor required for the gene expression response to RES. A brief exposure to excess light, which presumably generates ^1^O_2_*, H_2_O_2_, and possibly RES, induces acclimation to ^1^O_2_*. t-BOOH, *tert*-butyl hydroperoxide; SAK1, SINGLET OXYGEN ACCLIMATION KNOCKEDOUT 1; MBS, METHYLENE BLUE SENSITIVE; PSBP2, PHOTOSYSTEM II SUBUNIT P2; SOR1, SINGLET OXYGEN RESISTANT 1.

So far, transcriptional regulators that directly interact with a specific ROS have not been characterized in *Chlamydomonas*. On the other hand, *Chlamydomonas* is uniquely capable of sensing and acclimating to a low level of ^1^O_2_*, allowing cells to tolerate subsequent higher levels of ^1^O_2_* stress (Ledford et al., 2007), similar to the microbial ROS-specific responses discussed above. The disruption of the phosphoprotein SAK1 abolishes ^1^O_2_* acclimation in *Chlamydomonas.* The mutant *sak1* lacks the induction of the most strongly upregulated genes during acclimation to ^1^O_2_*, indicating the central role of SAK1 in this process (Wakao et al., 2014). A small zinc finger protein METHYLENE BLUE SENSITIVE is also necessary in *Chlamydomonas* and Arabidopsis for the induction of some ^1^O_2_*-responsive genes (Shao et al., 2013). A role for PHOTOSYSTEM II SUBUNIT P2 (PSBP2) in the response to ^1^O_2_* has been identified in a mutant screen using a ^1^O_2_*-responsive reporter line (Brzezowski et al., 2012). How or whether these proteins genetically and biochemically interact is still unknown (Figure 3).

^1^O_2_*- or H_2_O_2_-specific gene regulation has been studied for several individual genes in *Chlamydomonas*. In an early study, *GPX5* (also known as *GPXH*) was discovered as a gene that is strongly induced by ^1^O_2_* and moderately by RES (Leisinger et al., 2001), while it responds weakly to H_2_O_2_ or O_2_^−^. As with the other *Chlamydomonas* GPXs, GPX5 belongs to the phospholipid peroxidase family that can reduce oxidized FAs from the membrane (Dayer et al., 2008). GPX5 has been shown to use TRX rather than GSH as an electron donor to reduce H_2_O_2_ and alkyl peroxides (Fischer et al., 2009), and its promoter region contains motifs responsible for ^1^O_2_*- and cAMP-specific response (Leisinger et al., 2001). Different isoforms of GPX are predicted to reside in different subcellular compartments as ROS scavengers. In particular, expressed sequence tags suggest that *GPX5* has two alternative transcripts encoding enzymes that are differentially targeted to the chloroplast and cytosol (Dayer et al., 2008). The *HSP70* promoter has been dissected into regions that allow H_2_O_2_ and ^1^O_2_*-specific response, indicating the two inputs can independently or synergistically upregulate this gene (Shao et al., 2007). The induction of *VTC2* gene expression for ascorbate biosynthesis has been observed in response to ^1^O_2_* (Wakao et al., 2014) and H_2_O_2_ separately (Urzica et al., 2012; Blaby et al., 2015), hinting at the existence of independent or overlapping signaling pathways. In a proteomic study, Barth et al. (2014) separated responses that are ROS- or light-dependent by combining low versus high light intensity and aerobiosis (O_2_) versus anaerobiosis (N_2_) treatments. ROS-dependent differences were extracted by comparing proteins overrepresented in O_2_ versus N_2_ treatment at the same light intensity. Functions enriched in this group of proteins included ascorbate synthesis or its regeneration, including VTC4 (L-galactose 1-phosphate phosphatase), VTC1 (GDP-D-mannose pyrophosphorylase), and dehydroascorbate reductase (DHAR), resembling the ^1^O_2_*- or H_2_O_2_-dependent induction of *VTC2* gene expression. Conversely, a light-specific response was attributed to those proteins that were overrepresented in high light as compared to low light under anaerobiosis (eliminating ROS production). This group included proteins involved in the carbon-concentrating mechanism and nonphotochemical quenching (NPQ), indicating the induction of these proteins do not require ROS as an input. Some isoforms of enzymes involved in ROS metabolism such as TRX, peroxiredoxin, and glutaredoxin were found to require both light and ROS as input signals, and neither was sufficient on its own (Barth et al., 2014). The ROS-dependent response described in this study may be dominated by the response to H_2_O_2_ due to its stability and abundance.

Orthologs of the most strongly induced ^1^O_2_*-responsive genes in *Chlamydomonas* are not among those induced by ^1^O_2_* in Arabidopsis, suggesting that the two organisms respond to ^1^O_2_* in distinct ways at least at the transcript level (op den Camp et al., 2003; Ramel et al., 2012b; Wakao et al., 2014). Studies of the transcriptome dynamics during ^1^O_2_*-specific signaling in Arabidopsis have used several mutants: *flu*, deregulated in the accumulation of a photosensitizer, protochlorophyllide, in the dark (Meskauskiene et al., 2001); *ex1*, a suppressor of *flu* (Wagner et al., 2004), mentioned above; and *chlorina*, a chlorophyll *b*-less and thus LHCII-less mutant that overproduces ^1^O_2_* under lower light intensity as compared to the wild-type (Ramel et al., 2013). The global gene expression response to ^1^O_2_* in plants is distinct from that for H_2_O_2_ and O_2_^−^ and highly complex in Arabidopsis*.* An integral part of the response is the induction of lipoxygenases and synthesis of jasmonic acid, a phytohormone that governs part of the ^1^O_2_* response (op den Camp et al., 2003; Przybyla et al., 2008). In *Chlamydomonas*, ^1^O_2_*-specific transcriptome changes have been described during ^1^O_2_* acclimation, which revealed genes encoding transporters and enzymes involved in lipid metabolism (Wakao et al., 2014). The selective upregulation of genes with transporter functions during acclimation is consistent with the set of genes overexpressed in a mutant resistant to ^1^O_2_*, *singlet oxygen resistant 1* (*sor1*, discussed further below) in the absence of the exogenous ^1^O_2_*-generating photosensitizer, Rose Bengal (Fischer et al., 2012). This may imply that in *Chlamydomonas*, pumping out photosensitizers such as Rose Bengal is indeed part of the genetically programmed response against ^1^O_2_* unlike that of Arabidopsis. The gene expression response of *Chlamydomonas* also includes *GPX5* and genes annotated as cyclopropane fatty acid synthases (Wakao et al., 2014). Intriguingly, these genes are induced in response to ^1^O_2_* in an anoxygenic photosynthetic organism. The purple nonsulfur bacterium *Rhodobacter sphaeroides* shuts down photosynthesis in the presence of ^1^O_2_*, and its ^1^O_2_*-triggered transcriptional cascade is well described (Anthony et al., 2005; Ziegelhoffer and Donohue, 2009; Nam et al., 2013). Recently a furan fatty acid modification was found as an early event during the response of *R. sphaeroides* to ^1^O_2_* (Lemke et al., 2014, 2020). We are currently investigating whether similar fatty acid modification occurs in *Chlamydomonas* when exposed to ^1^O_2_*.

## Cross-talk between ROS and other reactive species

The ability of different RES, such as those containing α,β-unsaturated carbonyl groups, to induce signaling or gene expression has been known in plant defense responses (Améras et al., 2003; Farmer and Mueller, 2013). A *Chlamydomonas*^1^O_2_*-resistant mutant, *sor1*, constitutively overexpresses genes that are normally induced by several RES (Fischer et al., 2012). The mutation produces a dominant form of a bZIP transcription factor that activates RES-responsive genes and increases ^1^O_2_* tolerance, highlighting the overlap between physiological responses to ^1^O_2_* and to RES (Fischer et al., 2012). Intriguingly, a nontoxic low dose of the RES acrolein in wild-type *Chlamydomonas* cells increases resistance to subsequent ^1^O_2_* generated by Rose Bengal (Roach et al., 2018; Figure 3). This cross-acclimation resembles the resistance to RB-produced ^1^O_2_* that is elicited by a brief exposure to excess light (Ledford et al., 2007; Figure 3). Not surprisingly, genes that are upregulated by ^1^O_2_* overlap with those induced by acrolein (Roach et al., 2018).

The free radical nitric oxide (NO) and its cellular derivatives, such as nitrosoglutathione (GSNO) and peroxynitrite (ONOO^−^), can interact with cysteine or tyrosine residues and are collectively referred to as reactive nitrogen species (RNS) (Astier et al., 2021). NO has been described in various stress responses in *Chlamydomonas* and in other algae (reviewed by Astier et al., 2021). Strong light intensities (3,000 µmol photons m^−2^ s^−1^ in Chang et al., 2013; 1,600 µmol photons m^−2^ s^−1^ in Kuo et al., 2020b) have been associated with a simultaneous burst of NO and ROS (H_2_O_2_ and ^1^O_2_*) that coincides with cell death (Chang et al., 2013; Kuo et al., 2020b). The burst of NO is accompanied by a decrease in PSII electron transfer similar to what was observed in an earlier study in peas using NO inhibitors and donors (Wodala et al., 2008). The role of NO under a more physiological excess light condition remains to be examined. Plants and algae lack the animal-type NO signaling via cGMP, leaving protein nitrosylation as a main known route for regulation by RNS (Astier et al., 2021). A proteomic study of protein nitrosylation by GSNO has identified 492 S-nitrosylated proteins, including many involved in photosynthesis and other cellular processes but most notably, all CB cycle enzymes were found as nitrosylation targets (Morisse et al., 2014).

## Protein disulfide redox regulation

Redox regulation of protein function is usually focused on the reactivity of cysteine sulfhydryls, especially oxidation to form intramolecular, intermolecular, and mixed disulfides (e.g. glutathionylation), although other signals such as the redox state of the plastoquinone pool (Pfannschmidt et al., 1999) are also important in photosynthetic organisms. Here, we discuss protein disulfide redox regulation in *Chlamydomonas* that is linked to PSI via the ferredoxin/TRX system (Buchanan, 2016). A method using a TRX affinity column to trap potential target proteins via the formation of an intermolecular disulfide has been applied in many organisms, including *Chlamydomonas* (Lemaire et al., 2004). The list of *Chlamydomonas* proteins possibly targeted by TRX has expanded recently from 55 to ∼1,000 by combining this approach with mass spectrometry (Lemaire et al., 2004; Pérez-Pérez et al., 2017). The Lemaire group has compiled their redox proteome data and found that all 11 enzymes of the CB cycle are subject to glutathionylation (Zaffagnini et al., 2012), nitrosylation (Morisse et al., 2014), and Trx interaction (Pérez-Pérez et al., 2017). For some of these CB enzymes, glutathionylation has been shown to reversibly decrease the target enzyme activity under oxidative stress. Moreover, some cysteine residues on CB enzymes are shared targets of glutathionylation and nitrosylation, such as those on phosphoglycerate kinase and phosphoribulokinase (Michelet et al., 2013b). The observation of multiple redox PTMs on the same protein suggests complex layers of signaling and possible synergism or antagonism. For example, Cys178 of *Chlamydomonas* isocitrate lyase is targeted and inactivated by nitrosylation (Morisse et al., 2014) and glutathionylation (Bedhomme et al., 2009). Alternatively, glutathionylation and other PTMs occurring on the same Cys could be antagonistic, such as sulfenylation on DHAR2 (Waszczak et al., 2014) and nitrosylation on GAPC1 (Bedhomme et al., 2012) of Arabidopsis. However, outside of the CB cycle, the majority (∼70%) of proteins identified are subject to only one PTM, and among cysteines identified as redox regulated, 75% are sites of only a single modification (Pérez-Pérez et al., 2017).

Many proteins with redox-active cysteines are also phosphorylated. An earlier study had identified 4588 phosphorylation targets from *Chlamydomonas*, including two proteins already known to be subject to TRX regulation, sedoheptulose-1,7-bisphosphatase1, and phosphoribulokinase1; Wang et al., 2014). McConnell et al. (2018) directly investigated combinatorial protein phosphorylation and redox modification at cysteine residues by first capturing proteins with oxidized cysteine and then surveying their phosphorylation. A total of 1,457 proteins exhibited one or more oxidized cysteines; 720 were also phosphoproteins, and 172 of these contained cysteines that are subject to reversible oxidation and reduction (McConnell et al., 2018). The CB proteins sedoheptulose-1,7-bisphosphatase, phosphoribulokinase, CP12, and Rubisco activase were identified as proteins that are subject to regulation by both redox and phosphorylation, consistent with multiple layers of post-translational regulation of the CB cycle. These emerging large-scale data must be validated for each target, and further investigation is required to determine whether and how each PTM affects protein activity. An exciting direction in the field of PTM-focused proteomic studies will be to gain an integrative view, by examination of multiple PTMs under physiological conditions and identification of the target sites and their impact on protein function.

Calcium is another signal that is closely associated with ROS/redox signaling. In *Chlamydomonas*, the protein calredoxin was discovered to integrate two signals, Ca^2+^ and disulfide redox on a single chloroplast protein that contains two domains, Ca^2+^-sensing (four EF-hands) and TRX (Hochmal et al., 2016). In vitro, the calredoxin protein reduces peroxiredoxin to drive H_2_O_2_ detoxification, and a calredoxin mutant exhibits photooxidative stress (measured by elevated malondialdehyde and increased cyclic electron flow). Calredoxin putative orthologs are limited to green algae, and it is unknown why land plants lack orthologs and whether there are proteins that serve similar functions. The high-light induction of the major protein necessary for regulation of photosynthetic light harvesting by NPQ in *Chlamydomonas*, LHCSR3, requires the Ca^2+^-sensing protein CaS (Petroutsos et al., 2011), but this response does not seem to involve calredoxin as the mutant is only modestly reduced in LHCSR3 accumulation upon excess light (Hochmal et al., 2016).

## The cellular redox buffer: ascorbate–GSH cycle

To keep ROS under control, oxygenic photosynthetic organisms have invented the unique partnership of the universal redox molecules GSH and ascorbate (Asc) to maintain cellular redox homeostasis (reviewed by Gest et al., 2013). GSH and Asc are able to donate electrons to various oxidized compounds, and the perfect alignment of their redox potentials allows electrons to flow from NADPH, to GSH, and then to Asc (Foyer and Noctor, 2011). Phylogenetic analysis of the distribution of Asc biosynthetic and metabolic enzymes suggests that photosynthetic organisms decoupled Asc biosynthesis from H_2_O_2_ generation through evolution, which was crucial to Asc becoming a central player in photoprotection (Wheeler et al., 2015). On the other hand, this highly functional redox buffer comes with the constant need for regeneration (reduction) of Asc and GSH from their oxidized forms monodehydroascorbate (MDHA), dehydroascorbate (DHA), and glutathione disulfide (GSSG; Figure 1A; reviewed by Foyer and Noctor, 2011). No mutants in land plants or *Chlamydomonas* have been isolated that are devoid of either Asc or GSH, indicating that complete loss is lethal. It has been suggested that Asc was adapted as a highly versatile antioxidant and cofactor of various enzymes in plants as the concentration of oxygen increased in the atmosphere (Gest et al., 2013). Interestingly, the number of enzymes utilizing Asc and the cellular concentration of Asc increase alongside the evolution and divergence of cyanobacteria, green algae, and land plants. This has led to speculations that Asc may not have as critical or diverse roles in algae as compared to plants (Gest et al., 2013). However, a number of recent studies on the reducing enzymes of the Asc–GSH cycle and Asc biosynthesis have suggested that this is not the case, and this work has also revealed additional functions for Asc in *Chlamydomonas*.

*Chlamydomonas* strains that are silenced for one of the two GSH reductase (*GR*) genes, which are induced during excess light, have increased photosensitivity (Lin et al., 2018). Similarly, silencing of the enzymes DHAR and MDHA reductase (MDAR) that regenerate Asc from its oxidized forms, DHA and MDHA, respectively, results in enhanced photosensitivity (Lin et al., 2016). In a pattern fitting the term “redox buffer”, the synthesis of both GSH and Asc increases when the redox balance shifts to a largely oxidized state after transfer to higher light intensity, and it recovers several hours after transfer (Lin et al., 2016; Vidal-Meireles et al., 2017). Depletion of total GSH or Asc is observed in the strains silenced for GR, DHAR, and MDAR, after the cell experiences excess light stress (Lin et al., 2016, 2018). The overexpression of DHAR and MDAR results in the converse, with improved PSII efficiency (*F*_v_/*F*_m_), decreased lipid oxidation, and less cell death under excess light, indicating that enhancing Asc regeneration increases tolerance to excess light (Lin et al., 2016). Not surprisingly, the disruption of Asc biosynthesis has an analogous effect to the loss of MDAR and DHAR. The Arabidopsis mutant *vtc2*, disrupted in the rate-determining step in ascorbate biosynthesis, contains 10%–30% of WT ascorbate and shows chronic photooxidative stress (Conklin et al., 2000; Müller-Moulé et al., 2004). Ascorbate biosynthesis in plants and green algae including *Chlamydomonas* shares the same enzymes and intermediates, whereas the biosynthetic pathways in other algae such as *Euglena*, diatoms, and rhodophytes are distinct (Wheeler et al., 2015). This is reflected in the phenotypic similarities of the specific mutants found in Arabidopsis and *Chlamydomonas* and may suggest that the underlying regulatory mechanisms in the two organisms are shared as well. The silencing of the *VTC2* gene in *Chlamydomonas* similarly results in photosensitivity that is at least in part attributed to the defect in expanding the Asc pool upon stress (Vidal-Meireles et al., 2017). Multiple subcellular forms of APX have been implicated as critical enzymes in the excess light response in Arabidopsis (Karpinski et al., 1997; Awad et al., 2015; Exposito-Rodriguez et al., 2017). In *Chlamydomonas*, the silencing of *APX4*, the most strongly induced isoform upon excess light among the three *Chlamydomonas* genes encoding chloroplast APXs, resulted in photosensitivity (Kuo et al., 2020a). Similar to strains silenced for Asc biosynthesis or reduction, *apx4* silenced lines show a highly oxidized Asc pool under excess light. These studies collectively highlight the importance of Asc and the interconnected relationship between ascorbate biosynthesis, H_2_O_2_ detoxification-coupled Asc oxidation, and Asc regeneration. Perturbation of any part of the finely tuned cycle results in the oxidation and depletion of the Asc–GSH redox buffer and ultimately causes an irreversible oxidative burst in the cell (Figure 2, red zone).

During evolution, Asc has acquired photoprotective functions beyond serving as an antioxidant (Gest et al., 2013). In plants, ascorbate is the electron donor for violaxanthin de-epoxidase (VDE; Hager, 1969; Muller-Moule et al., 2002), an enzyme required for the formation of zeaxanthin, the xanthophyll pigment involved in NPQ (reviewed by Demmig-Adams, 1990). However, the *Chlamydomonas vtc2* mutant with strongly decreased Asc exhibits normal de-epoxidation of violaxanthin (Vidal-Meireles et al., 2020), in contrast to the situation in the *vtc2* mutant of Arabidopsis (Müller-Moulé et al., 2004). *Chlamydomonas* lacks a plant-type VDE and instead uses an unrelated enzyme called Chlorophycean VDE (CVDE) that is annotated as aflavin adenine dinucleotide (FAD)-dependent oxidoreductase (Li et al., 2016b), and thus it does not require Asc for de-epoxidation of violaxanthin to zeaxanthin in vivo (Vidal-Meireles et al., 2020). The *Chlamydomonas vtc2* mutant displays a slowly relaxing NPQ that is enhanced by H_2_O_2_ and reversed by the addition of catalase (Vidal-Meireles et al., 2020). These results are consistent with those from Arabidopsis indicating that the cause of photosensitivity in the *vtc2* mutant is elevated H_2_O_2_ and other ROS, rather than disruption of the xanthophyll cycle and NPQ (Müller-Moulé et al., 2004; Vidal-Meireles et al., 2017, 2020). In contrast to *Chlamydomonas*, many other green algae contain a plant-type VDE (Coesel et al., 2008) that likely catalyzes the Asc-dependent formation of zeaxanthin as shown in *Chlorella vulgaris* (Girolomoni et al., 2020). A gene encoding a VDE-related enzyme (VDR) is broadly conserved in algae and plants (Coesel et al., 2008; Karpowicz et al., 2011; Girolomoni et al., 2020), but its biochemical function has not been elucidated. Another related enzyme of this family, VDE-like (VDL), has been found to catalyze the Asc-independent formation of cis-neoxanthin, a precursor of the major marine algal carotenoid, fucoxanthin (Dautermann et al., 2020).

Recent studies have implicated Asc in an interesting *Chlamydomonas*-specific function in which it inactivates PSII. Ascorbate is capable of donating electrons to PSII when PSII is inactivated, for example during heat stress and prolonged darkness in plants (Tóth et al., 2009; Podmaniczki et al., 2021). In sulfur-deprived *Chlamydomonas* cells, Asc levels increase 50-fold, and in the millimolar range Asc interacts with and inactivates the Mn cluster in the oxygen-evolving complex of PSII (Nagy et al., 2016). The induction of Asc biosynthesis seems to involve ^1^O_2_*, which is generated during sulfur deprivation (Nagy et al., 2018). The inactivation of PSII facilitates the establishment of anaerobiosis, and Asc continues to donate electrons to sustain H_2_ production, demonstrating a function of Asc in preventing the production of O_2_ and ROS at unusually high cellular concentrations of Asc (Nagy et al., 2016).

Asc was found to stimulate DNA methylation in mouse embryo through the activity of ten-eleven translocation (TET) dioxygenase that oxidizes 5-methyl cytosine to 5-hydroxy methylcytosine, which then promotes global demethylation (Blaschke et al., 2013). A mutant in a TET dioxygenase-like protein in *Chlamydomonas* revealed a DNA modification, 5-glyceryl methylcytosine that is produced using a glyceryl moiety directly from Asc (Xue et al., 2019). The mutant genome exhibits increased DNA methylation, including the *LHCSR3* locus, coinciding with the attenuation of its excess light induction at the transcript and protein level (Xue et al., 2019). The glycerylation of 5mC is speculated to lead to other epigenetic modifications that may act antagonistically against further methylation and silencing of the respective locus. These findings present examples of additional functions for which *Chlamydomonas* utilizes Asc to regulate photoprotection.

## Summary

The unicellular alga *Chlamydomonas* has served as a powerful experimental model for studying PTM and gene expression by proteomics and transcriptomics, respectively. Naturally these large-scale data present many interesting hypotheses that can be tested individually in the future. *Chlamydomonas* has been a powerful model organism for genetic studies of photosynthesis starting with the classical acetate-requiring mutants (Levine, 1960) to the more recently described, whole-genome sequenced Acetate-Requiring Collection (Wakao et al., 2021) and the large-scale Chlamydomonas Library Project (Li et al., 2016a, 2019). The time is ripe for testing and discovering gene functions with the wide availability of mutants and the CRISPR-Cas9 genome-editing technology that has been steadily improving in *Chlamydomonas*. The abundance of omics data also allows broader examination to identify overlapping pathways versus those that are unique to specific treatment or physiological condition (comparative proteomics or transcriptomics). An integrated understanding of the individual responses (e.g. from each modification on single proteins to the effect on an entire pathway consisting of multiple enzymes targeted by multiple modifications) within a physiological condition such as excess light would be an ultimate goal. *Chlamydomonas* continues to prove its strength as a photosynthesis research model, but it is also revealing unique aspects in its ^1^O_2_* signaling and responses, molecular functions of Asc, and calredoxin. With more green algal genomes becoming available and the advancement of computational methods in identifying orthologous genes, what we now describe as *Chlamydomonas-*specific response may serve as the starting point for understanding of more widespread responses to ROS and redox in the green lineage (see Outstanding Questions Box).

**ADVANCES BOX**

- Proteomic studies focused on redox-thiol modifications indicate that the majority of the enzymes in the CB cycle are targeted by more than one post-translational modification.
- The genes induced by ^1^O_2_* in *Chlamydomonas* overlap with those induced by RES, and pre-exposure to RES acclimates cells to ^1^O_2_*, indicating that one of the products of ^1^O_2_* oxidation (i.e. RES) is also a critical player in the gene expression response to ^1^O_2_*.
- In *Chlamydomonas*, ascorbate has functions in photoprotection that have not been previously described in plants, including DNA modification and suppression of ROS production through inactivation of PSII.

**OUTSTANDING QUESTIONS**

- What are the physiological conditions under which the protein redox-thiol modifications occur?
- How do co-occurring post-translational modifications affect each target protein? Do the multiple modifications affect function independently, or do they have a synergistic effect? And how does that impact the overall activity of the pathway?
- What are the regulatory players in ROS signaling and gene expression for both ROS-specific and overlapping responses?
- Are some of the recently discovered functions of ascorbate in *Chlamydomonas* shared in plants and/or other green algae?

## Notes

The author responsible for distribution of materials integral to the findings presented in this article in accordance with the policy described in the Instructions for Authors (https://academic.oup.com/plphys/pages/General-Instructions) is: Krishna Niyogi (niyogi@berkeley.edu).
